# Use of Micellar Delivery Systems to Enhance Curcumin’s Stability and Microbial Photoinactivation Capacity

**DOI:** 10.3390/foods10081777

**Published:** 2021-07-31

**Authors:** Victor Ryu, Silvette Ruiz-Ramirez, Piyanan Chuesiang, Lynne A. McLandsborough, David Julian McClements, Maria G. Corradini

**Affiliations:** 1 Department of Food Science, University of Massachusetts Amherst, Amherst, MA 01003, USA; vryu@foodsci.umass.edu (V.R.); silvette.ruiz@upr.edu (S.R.-R.); mcclements@foodsci.umass.edu (D.J.M.); 2 Department of Food Technology, Faculty of Science, Chulalongkorn University, Bangkok 10330, Thailand; pchuesiang@umass.edu; 3 Food Science Department and Arrell Food Institute, University of Guelph, Guelph, ON N1G 2W1, Canada

**Keywords:** microbial photoinactivation, curcumin, micelle, photosensitizer, reactive oxygen species, singlet oxygen (^1^O_2_), critical micelle concentration, *Escherichia coli*, *Listeria innocua*

## Abstract

Microbial photoinactivation using ultraviolet (UV) or visible light can be enhanced by photosensitizers. This study assessed the efficacy of encapsulating a food-grade photosensitizer (curcumin) in surfactant micelles on its water dispersibility, chemical stability, and antimicrobial activity. Stock curcumin-surfactant solutions were prepared with Surfynol 465 (S465) or Tween 80 (T80) (5 mM sodium citrate buffer). The antimicrobial activity of curcumin-loaded surfactant solutions was determined by monitoring the inactivation of *Escherichia coli* O157: H7 and *Listeria innocua* after 5-min irradiation with UV-A light (λ = 365 nm). The solutions mixed with the bacterial suspensions contained 1 µM curcumin and each surfactant below, near, and above their critical micelle concentrations (CMCs). The addition of surfactants at any level to the curcumin solution enhanced its dispersibility, stability, and efficacy as a photosensitizer, thereby enhancing its antimicrobial activity. Gram-positive bacteria were more susceptible than Gram-negative bacteria when curcumin-loaded micelles were used against them. The photoinactivation efficacy of curcumin-surfactant solutions depended on the pH of the solution (low > high), surfactant type (S465 > T80), and the amount of surfactant present (below CMC ≥ near CMC > above CMC = unencapsulated curcumin). This result suggests that excessive partitioning of curcumin into micelles reduced its ability to interact with microbial cells. Synergistic antimicrobial activity was observed when S465 was present below or near the CMC with curcumin at pH 3.5, which could be attributed to a more effective interaction of the photosensitizer with the cell membranes as supported by the fluorescence lifetime micrographs. The use of a micelle-based delivery system facilitates adsorption and generation of reactive oxygen species in the immediate environment of the microbial cell, enhancing photoinactivation.

## 1. Introduction

Effective sanitation of food-contact surfaces reduces the risk of contamination of food products with pathogenic or spoilage microorganisms [1]. Chlorine compounds are one of the most common chemical sanitizers used on food surfaces within the food industry. The Food and Drug Administration (FDA) recommends that food processing equipment and food-contact surfaces are sanitized with no more than 200 parts per million (ppm) of available chlorine [2]. Application of chlorine above the recommended levels can result in excessive residual chlorine that could potentially impart adverse flavors and odors to food products or cause a safety hazard if the sanitizing solution is exposed to acids due to the production of chlorine gas [2]. Although, prior to sanitizating in the food industry, a cleaning stage is carried out, the effectiveness and safety of chlorinated water as a sanitizer could also be compromised by the presence of remaining organic matter on the surface due to the rapid depletion of chlorine and the formation of by-products such as trihalomethanes, which are carcinogenic [3,4,5]. Alternative sanitation methods for contact surfaces have, therefore, been proposed, such as ozone, organic acids, and essential oils. However, concerns about their limited efficacy (reductions of only 1–3 log_10_ cycles), environmental impact, ability to generate harmful and persistent by-products, and/or to promote the emergence of microbial resistance have precluded their widespread utilization [6].

Photodynamic inactivation involves the combined use of ultraviolet (UV) or visible light and photosensitizers (PS). Photoinactivation of microorganisms on food surfaces using a solution of photosensitizers and an available light source could result in an effective, environmentally friendly, and safe alternative to current sanitizers. To comply with these requirements, the photosensitizer solution needs to be stable, easy to prepare, and effective, even after storage. Additionally, none of the components of the solution should be toxic, produce harmful by-products or leave undesirable residues, which can be attained by using natural products that behave as photosensitizers at low concentrations.

**Figure 1:**
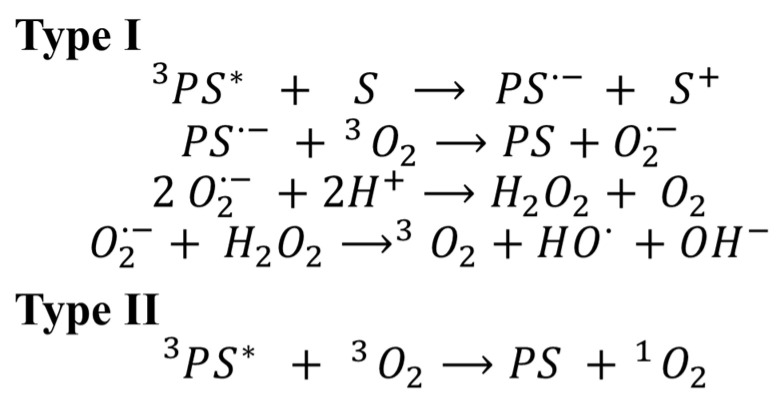
Type I & II photoreactions involved in the generation of ROS. (H_2_O_2_ is hydrogen peroxide and OH^−^ is hydroxide.).

Photosensitizers have the ability to absorb energy from light and then transfer it to acceptor molecules, such as a surrounding substrate (S) or molecular oxygen (^3^O_2_). When the photosensitization process involves the interaction of a photosensitizer in its excited singlet (^1^PS*) or triplet state (^3^PS*) with the surrounding substrate, it is dubbed Type I [7,8]. This interaction results in the transfer (donation or reception) of electrons or protons from the photosensitizer to the surrounding substrate, and subsequent generation of radical anions that react with oxygen to form reactive oxygen species (ROS), such as superoxide anion (O_2_^•-^) and hydroxyl radicals (HO^•^) [9,10]. In Type II photoreactions, the photosensitizer in its triplet excited state (^3^PS*) collides with molecular oxygen, transferring its energy and generating excited singlet oxygen (^1^O_2_) [7,11] Characteristic Type I and II photoreactions, and the corresponding ROS generated by each of them, are presented in Figure 1.

The generated ROS (hydroxyl radicals or ^1^O_2_) can then inactivate pathogenic microorganisms in foods or food surfaces by damaging their cell membranes, organelles, and/or nucleic acids. Although both Type I and II reactions can occur simultaneously, most of the antimicrobial activity resulting from photosensitization processes is attributed to Type II reactions and the high reactivity of singlet oxygen (^1^O_2_) with cellular components such as lipids, proteins, and nucleic acids [12,13]. Singlet oxygen’s high reactivity is due to its π antibonding electrons in a single orbital [13]. Therefore, it quickly reacts with non-radical, singlet state, and electron-rich compounds containing double bonds [8]. It should be noted that singlet oxygen has a very short lifetime, typically lower than 3.5 microseconds, due to its fast radiative decay to the ground triplet state with emission at 1270 nm [14]. The short lifetime and high reactivity of singlet oxygen restrict the distance it can travel to a spherical domain of around 10 to 100 nm from where it was generated [13,15]. Furthermore, the lifetime of singlet oxygen is reduced in aqueous media due to the transfer of energy to the oxygen-hydrogen stretching mode of water molecules, which has a similar energy to that of singlet oxygen [13]. Thus, it is believed that photosensitizers must be close to or physically interacting with their target (e.g., microbial surfaces) to be effective. This is supported by research in photodynamic therapy and photodynamic inactivation, where photosensitizer uptake by mitochondria, lysosomes, organelles, or pathogenic bacteria was shown to increase treatment efficiencies [7,16,17]. We hypothesize that encapsulating a photosensitizer in a well-designed delivery system would improve its ability to interact with bacteria, thereby increasing its efficacy as an antimicrobial agent.

Curcumin was used in this study as a natural food-grade photosensitizer. Curcumin is the most bioactive and abundant compound present in *Curcuma longa* species [18]. However, it has a relatively low water solubility, i.e., 3.12 mg/L at 25 °C [19], limiting the amount that can be incorporated into aqueous solutions. Moreover, it tends to chemically degrade when exposed to light and can either precipitate or chemically degrade during storage, depending on the pH of the solution [20]. Curcumin is in a hydrophobic non-charged form in aqueous solutions from pH 1 to 7, which causes it to nucleate and precipitate out of solution. Conversely, it is partially charged from pH 7 to 10, which increases its hydrophilicity and water solubility but decreases its chemical stability. Indeed, curcumin has been shown to degrade into ferulic acid and ferulymethane under alkaline conditions [18]. Despite these shortcomings, curcumin has been used as an effective photosensitizer. For example, de Oliveira et al. [21] reported that curcumin (1–10 mg/L) reduced *E. coli* O157:H7 and *L. innocua* counts on fresh-produce surfaces by more than 5 log cycles at low pH in combination with UV-A light. The efficiency of curcumin as a photosensitizer could be enhanced by identifying an appropriate delivery system to mitigate potential problems, such as precipitation, and optimize its performance as a food-grade sanitizing agent. A desirable delivery system for photodynamic inactivation should meet the following criteria: (i) it should not increase light scattering (e.g., low turbidity), as photoactivation and, consequently, ROS generation depends on light penetration; (ii) it should not absorb light at the same wavelength as the encapsulated photosensitizer; (iii) it should promote the partitioning of the encapsulated photosensitizer into the pathogenic bacteria; and (iv) all of its components should be approved for use on food-contact surfaces.

Based on these requirements, micellar delivery systems would appear to be highly suitable for encapsulating curcumin so as to increase its efficacy as a photosensitizer and its antimicrobial activity against bacteria. Surfactant micelles consist of small particles that are spontaneously assembled from surfactant molecules due to hydrophobic effects [22]. These colloidal particles have a hydrophobic interior that is capable of solubilizing non-polar molecules such as curcumin, as well as a hydrophilic exterior that allows them to be readily dispersed within water. Surfactant micelles are thermodynamically favorable systems, which means that once formulated, they should remain stable indefinitely. Micelles tend to form when the surfactant concentration in solution exceeds the critical micelle concentration (CMC). Below the CMC, the surfactant molecules are dispersed as monomers in water, but above the CMC, surfactant micelles and monomers co-exist. Previous studies have reported that micelles could be used to encapsulate and stabilize curcumin [23,24,25]. The dimensions of surfactant micelles (<20 nm) are typically much lower than the wavelength of light (380–780 nm), which means that they only scatter light very weakly, and the solution appears transparent. This is an advantage for the development of delivery systems for photosensitizers because it means that more light penetrates into the interior of the system, thereby increasing photoexcitation of the photosensitizer and ROS generation [26]. Also, as a result of their small particle size, micelles move rapidly due to Brownian motion, which increases their interactions with bacteria. Finally, micelles are highly dynamic systems, which means that curcumin molecules may be rapidly exchanged between the hydrophobic interiors of the micelles and cell membranes in the bacteria.

This study examined the impact of encapsulating curcumin within surfactant micelles on its water dispersibility, chemical stability, and antimicrobial efficacy. Two surfactants approved for food contact surface applications were used to assemble the micelles: Surfynol 465 and Tween 80. The physicochemical and structural properties of the curcumin-loaded surfactant micelles were characterized, and their photoinactivation capacity was evaluated against two model food pathogens, *Escherichia coli* O157:H7 and *Listeria innocua*. The results of this study may lead to the development of more efficacious antimicrobial formulations to treat food-contact surfaces.

## 2. Materials and Methods

### 2.1. Materials

Curcumin with a purity higher than 97% was purchased from TCI Chemicals (C2302-5G, Montgomeryville, PA, USA). Two nonionic surfactants, Surfynol 465 (S465) and Tween 80 (T80), were obtained from Shenzhen Vtolo Industrial Co. Ltd. ( Shenzhen, Guangdong, China) and Sigma-Aldrich (P1754, St Louis, MI, USA), respectively. 5 mM sodium citrate buffer was prepared using citric acid monohydrate (C7129, Sigma-Aldrich, St. Louis, MO, USA) and sodium citrate (#775538, Fisher-Scientific, Waltham, MA, USA). Absolute ethanol (#111000200) was obtained from Pharmco (Brookfield, CT, USA).

### 2.2. Stock Curcumin-Surfactant Solutions

A curcumin stock solution (4 mM) was prepared by dissolving curcumin into ethanol. Surfactant solutions were prepared by dissolving different amounts of S465 or T80 in 5 mM sodium citrate buffer at pH 3.5 and stirring at 125-rpm for 20 min. The curcumin stock solution was titrated (2.5 mL/min) into each surfactant solution to produce 20 µM curcumin-surfactant stock solutions. After titration, the stock curcumin-surfactant solutions were sheared for 15 min. These conditions are known to induce nucleation and crystallization of curcumin under conditions where there is insufficient surfactant to fully solubilize the curcumin [20]. All the curcumin micellar stock solutions were then filter-sterilized using a 0.45 µm syringe filter (Cat# 02915-22, Cole-Palmer, Vernon Hills, IL, USA) and stored at 4 or 20 °C. The initial concentration of curcumin and surfactant in each stock solution was selected so that curcumin-surfactant systems containing 1 µM curcumin and surfactant concentrations below, at, or above the CMC could be obtained after dilution.

### 2.3. Maximum Curcumin Loading Capacity of Surfactant Solutions

The maximum curcumin loading capacities of surfactant solutions prepared at concentrations below, near, and above their CMCs were determined by titrating (2.5 mL/min) an alcoholic curcumin solution into each aqueous surfactant solution. The endpoint, taken to be the maximum loading capacity, was identified as the curcumin concentration that first caused an appreciable rise in turbidity, which was determined by measuring the absorbance at 600 nm after the samples had been stored overnight.

### 2.4. Stability of Stock Curcumin in Surfactant Solutions during Storage

Curcumin stability in the stock solutions was monitored during storage in a 4 or 20 °C incubator for 30 days. Visual observation was carried out by tilting the tube to a 45° angle for any evidence of crystallization every two days. The mean particle diameter (Z-average) and polydispersity index (PDI) of the micelles formed were measured using dynamic light scattering (Zetasizer Nano Z.S., model ZEN 3600, Malvern Instruments, Malvern, UK). The particle size distribution was calculated from the back-scattered laser intensity fluctuations using the instrument’s software (Malvern Zetasizer DTS software, Version 7.01).

The absorption of encapsulated curcumin over 30 days was determined using a UV-visible spectrophotometer (Shimadzu Scientific, Kyoto, Japan) at 425 nm in quartz cuvettes (1 cm path length). The size of the spectrophotometer slit was set at 1 nm. The intensity of the curcumin autofluorescence was also used to monitor curcumin solubility inside the micelle and the effect of surfactants on the photophysical properties of the solution using a spectrofluorometer (Fluoromax-4, Horiba Scientific Inc., Edison, NJ, USA). Fluorescence spectra of curcumin in stock surfactant solutions were recorded using an excitation wavelength of 365 nm over an emission range of 375 to 600 nm. The excitation and emission slits used were 2 and 3 nm, respectively.

### 2.5. Surface Potential of the Curcumin Micelles in the Stock Solutions

The net charge (ζ-potential) of the micellar solutions was measured using a particle electrophoretic light scattering instrument (Zetasizer Nano Z.S., model ZEN 3600, Malvern Instrument, Malvern, UK). Changes in ζ-potential were monitored during storage in a 4 or 20 °C incubator for 30 days.

### 2.6. Bacterial Culture Conditions

*E. coli* O157:H7 (ATCC-43888, non-toxigenic strain) and *L. innocua* Seelinger (ATCC-51742) were obtained from American Type Culture Collections (Manassas, VA, USA) to be used as surrogates of representative Gram-negative and Gram-positive foodborne pathogens. A frozen stock *E. coli* O157:H7 suspension was prepared in a mixture of tryptic soy broth (TSB; Cat# DF0064-07-6, B.D. Diagnostic Systems, Berkshire, UK) containing 25% *v/v* glycerol. For *L. innocua* Seelinger, the stock was prepared in a mixture of tryptic soy broth with 0.01% yeast extract (TSBYE) and 25% *v/v* glycerol. Both stocks were stored at −80 °C. A working stock was prepared by inoculating a loopful of frozen stock in TSB then incubating at 37 °C overnight. Subsequently, the working stocks were streak plated onto MacConkey Sorbitol Agar (Cat# 279100, B.D. Diagnostic Systems) or Modified Oxford Agar (Cat# 222530, B.D. Diagnostic Systems), which was then stored at 4 °C for a week.

For the photoinactivation assay, a colony was inoculated into TSB or TSB + 0.01%YE and incubated at 37 °C for 18 h on a 125-rpm shaker. For each experiment, 18 h cultures were prepared, and their optical density at 600 nm (OD600) was adjusted to 0.15 cm^−1^, which confirmed they initially contained approximately 9 log CFU/mL. Bacteria were then washed two times with phosphate-buffered saline (PBS) solution by centrifugation at 3 *g* for 3 min. OD600 was obtained again after washing to confirm that there was no significant loss of bacteria. The bacterial counts were confirmed by dilution and plating on Tryptic Soy Agar (TSA; Systems Cat# 236920, B.D. Diagnostic Systems) using the spread plate method. The culture was then used to prepare the samples to be photoinactivated.

### 2.7. Bacterial Photoinactivation

The photoinactivation of the selected bacteria, expressed as log reductions (i.e., log_10_ *[N*_0_*/N(t)],* where *N*_0_ is the initial inoculum count and *N*(*t*) is the count at time t after each treatment was used to quantify the antimicrobial activity of the different curcumin-surfactant systems. Samples were prepared by replacing a volume of buffer with different volumes of curcumin-surfactant stock solution to obtain a series of samples that had the same curcumin concentration (1 µM) but different surfactant concentrations (below, near and above the CMC). After the curcumin was incorporated, the bacteria were added to obtain 6 log CFU/mL. 2mL of each diluted solution, which was aliquoted into 4 wells in sterile 24-well non-treated plates (Celltreat^®^ #229524, Celltreat Scientific Products, Pepperel, MA, USA), and then stored in the dark for 5 min prior to treatment. After that, the samples were either incubated in the dark or irradiated for 5 min. The irradiated samples were treated using an XL-1500 UV-crosslinker (Spectronic Corporate, Westbury, NY, USA) equipped with UV-A light (λ = 365 nm). Inside the irradiation chamber, the 24-well plate was placed on an elevated platform 9 cm away from the light source. For each run, four wells were used to irradiate the samples. The position of the four wells was adjusted to expose all the wells to an irradiance of 5.2–5.4 μW/cm^2^. Verification of the irradiance and temperature in the wells were made with a UV A/B light meter (#850009, Sper Scientific, Scottsdale, AZ, USA) and a four-channel data logger (#800024, Sper Science), respectively. Controls for the independent effect of the UV light, each surfactant concentration without curcumin, and non-encapsulated curcumin on microbial inactivation were also run.

Bacterial survival after treatment was determined using the table for 3-tube most probable number (MPN) at 95% confidence interval, as described in the Food and Drug Administration Bacteriological Analytical Manual [27]. The MPN method was selected since its limit of detection allows monitoring photoinactivation efficacy even when the procedures caused a significant reduction in the counts of treated samples. This method was used to evaluate all the treatments, except for the effect of curcumin concentration on photoinactivation. After irradiation, samples were serial diluted in PBS (pH 7.4), and 1 mL from each dilution (10^−1^, 10^−2^, 10^−3^ or higher) was inoculated into triplicate wells containing 2 mL of TSB or TSBYE (for *E. coli* or *L. innocua*, respectively) in a sterile 24-well plate. After incubation at 37 °C for 24 h, the turbidity of the samples in each well was assessed visually as a presumptive positive growth. Confirmation was undertaken by streaking on selective media for each microorganism (i.e., MacConkey Agar (BD 211393) and Modified Oxford Agar (BD 222510) for *E. coli* and *L. innocua*, respectively). Confirmed positive wells were used for calculating the MPN/mL.

### 2.8. Fluorescence Lifetime Imaging Microscopy (FLIM)

Fluorescence Lifetime Imaging Microscopy (FLIM) was used to obtain information about the local environments of the intrinsic lumiphore (curcumin) in the studied systems, which provided insights into the partitioning of this photosensitizer into the microbial cells [28]. Bacteria were deposited onto a 35 mm poly-D-lysine-coated dish (P35GC-1.5-10-C, MatTek Life Sciences, Ashland, MA, USA), dried for 2 h under air circulation in a biological safety-hood, and then treated with 5 µM curcumin and S465 or T80 (near C.M.C.). The fluorescence lifetime measurements of the samples were performed using a Nikon TiE stand with an A1 Spectral Detector confocal microscope (Nikon Instruments Inc., Melville, NY, USA) equipped with a FLIM/Fluorescence Correlation Spectroscopy (FCS) module. Briefly, curcumin fluorescence was first imaged using the traditional scanning confocal capability of the microscope. Once a suitable field of view was found and focused, the input lasers were changed to a 405 nm pulsed laser, and the fluorescent photons produced were directed to the Becker-Hickl SPC-152/HPM-100-40 dual detector system (Boston Electronics). Curcumin fluorescence lifetime was measured using 50 Mhz pulse frequency. The recorded fluorescence decay curves were analyzed with Becker & Hickl SPC Image-fitting software was used to yield the mean fluorescence lifetime of 256 × 256 pixel lifetime images and characterized the fluorescence lifetime using a multi-exponential model (2 components).

### 2.9. Data Acquisition and Analysis

All experiments were carried out as three independent experimental replicates. The Statistical Package for the Social Sciences (SPSS, Version 25, SPSS Inc., Chicago, IL, USA) was used to perform the statistical analysis. A paired *t*-test was used to analyze differences in the average diameter of micelles, polydispersity (PDI), and antimicrobial efficacy of the curcumin-loaded micelles at days 0 and 30. This statistical test was also used to analyze differences in the average log reductions obtained in the irradiated and non-irradiated samples. Duncan’s new multiple-range tests were used to identify differences between averages of the diameter of micelles, PDI, and log reductions obtained at different conditions. All tests were performed using *p* ≤ 0.05 to represent a statistical significance.

## 3. Results and Discussion

### 3.1. Influence of pH on the Photoinactivation Capacity of Unencapsulated Curcumin

**Figure 2:**
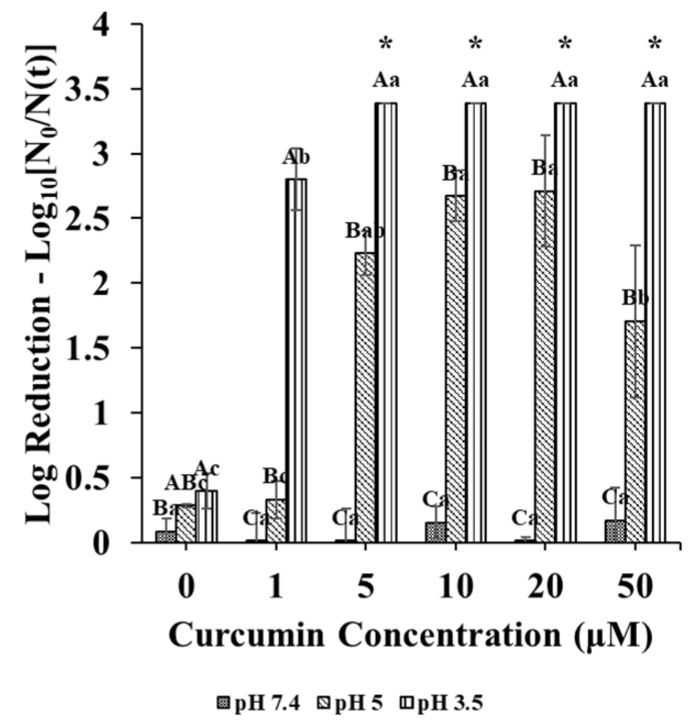
Reduction in *E. coli* O157: H7 from photoactivation with varying curcumin concentrations at pH 3.5, 5, or 7.4. “*” indicates that the remaining log count was below the limit of detection of the method (spread plating). Capital letters indicate differences between averaged log reductions at different pHs; samples with the same letters are not significantly different (*p* > 0.05). Lower-case letters indicate differences in averaged log reductions at different curcumin concentration; means with same letters are not significantly different (*p* > 0.05).

Initially, the antimicrobial activity of non-encapsulated curcumin was evaluated in different buffer solutions (pH 3.5–7.4) containing 6 Log CFU/mL of *E. coli* O157:H7 placed in a multi-well plate. Inoculated microbial suspensions (2 mL) containing different curcumin concentrations (0–50 µM) were irradiated with UV-A light (λ = 365 nm) for 5 min. As the pH of the buffer decreased, the antimicrobial efficacy increased (Figure 2). According to Tønnesen and Karlsen [18], the half-life of curcumin in aqueous solutions (pH 1.2–7.0) was longer at lower pH values. Thus, a higher concentration of curcumin remained in an active form at pH 3.5 than at pH 7.0. Additionally, this lower pH also might have contributed to a greater influx of non-dissociated citric acid into the microbial cells, thereby lowering the intracellular pH of the bacteria and enhancing inactivation, as reported by de Oliveira et al. [21]. At pH 3.5, the photoinactivation efficacy increased as the concentration of curcumin increased (Figure 2). At pH 5, the same photoinactivation trend was observed until the curcumin concentration reached 10 µM, then it decreased with a further increase (Figure 2). This effect may have been due to either an increased absorbance of light by the curcumin molecules or an increased scattering of light by any curcumin crystals formed at high concentrations, which interfere with photoactivation. At pH 7.4, photoinactivation by curcumin was adversely affected, probably due to rapid precipitation or degradation of the curcumin at this pH (Figure 2).

The antimicrobial assay employed in these experiments indicated that 1 µM of curcumin could inactivate at least 2.5 Log CFU/mL of *E. coli* O157:H7 at pH 3.5. Higher concentrations of curcumin at pH 3.5 gave count levels below the spread plate limit of detection. Therefore, 1 µM of curcumin was used in subsequent antimicrobial assays to elucidate the role of the delivery system on the efficacy of the photoinactivation process and the MPN method was used for further monitoring of the inactivation efficacy.

### 3.2. Influence of Surfactant Level on Curcumin Stability in the Stock Solutions

The maximum loading capacity of the S465 and T80 solutions was determined by titrating different amounts of curcumin into them and then measuring their turbidity. These experiments showed that T80 was more efficient at encapsulating curcumin than S465 (Figure S1-Supplementary Material). The molecular dimensions of surfactants are known to affect the shape and structure of the micelles formed [22], which impacts their solubilization properties. T80 and S465 have different molecular dimensions and polarities, as reflected by differences in their hydrophilic-lipophilic balance (HLB) values [29]. T80 has a hydrophilic polyoxyethylene head-group with an unsaturated hydrophobic tail with a kink and an HLB value of 15 [30]. In contrast, S465 exhibits a “Gemini” shape with two hydrophobic tails, two ethoxylated hydrophilic groups, and an HLB value of 13 [31]. These results are consistent with the results of Ma et al. [32] that reported a more uniform distribution and stability of curcumin in micellar systems prepared from surfactants with high HLB values. Based on these results, stock curcumin-surfactant solutions containing 20 µM of curcumin were produced using the two surfactants. This curcumin concentration was used because it was well below the maximum loading capacity of the curcumin for the two surfactants used. 

Three surfactant levels were used to produce the 20 µM curcumin-surfactant stock solutions: 92, 184, and 276 mM for S465 and 0.072, 0.24, and 0.36 mM for T80. The CMC of pure S465 and T80 have been reported to be around 10.5 mM or 11–16 mM [33,34] and 0.014 mM [25], respectively. Hence, a 1:20 dilution of the stock solution allows curcumin-surfactant solutions to be produced with surfactant concentrations below, near, and above the CMC. It should be noted that the CMC of a surfactant solution is affected by the presence of encapsulants and may either increase or decrease, depending on the molecular characteristics of the encapsulant [25]. 

**Figure 3:**
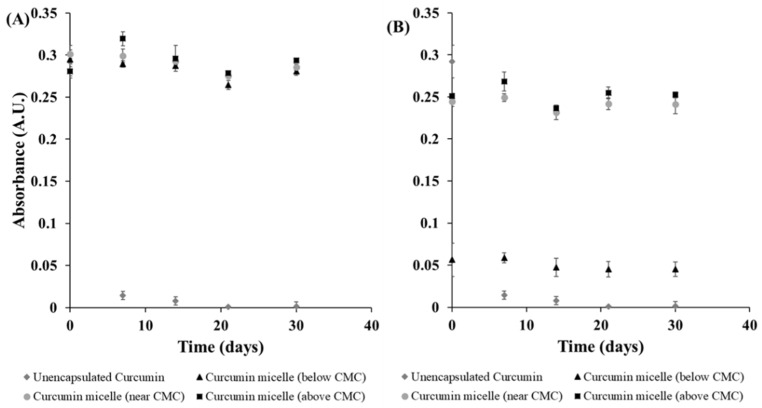
Stability of curcumin in solution and encapsulated in (**A**) S465 and (**B**) T80 at different concentrations during storage at 20 °C for 30 days. The data shows changes in the absorbance at 425 nm over time.

**Figure 4:**
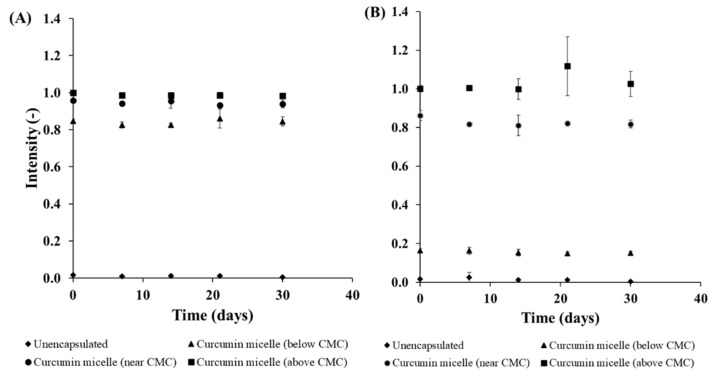
Normalized fluorescence-emission intensity of curcumin in solution and encapsulated in (**A**) S465 and (**B**) T80 at different concentrations during storage at 20 °C for 30 days (λ_exc_ = 365 nm, λ_em_ = 500 nm).

Changes in the solubility and stability of curcumin during storage were assessed by measuring changes in the absorbance and emission intensity of the diluted curcumin-surfactant solutions. The diluted solutions were within the linear region of the intensity versus concentration relationship, thereby avoiding any inner-filter effects that can cause problems for fluorescence measurements. In contrast to the pure curcumin solutions, the presence of either S465 and T80 prevented nucleation and precipitation of the curcumin, as reflected by the lack of a reduction in absorbance and fluorescence intensity values during storage for 30 days at 4 or 20 °C (Figure 3 and Figure 4, Figures S2 and S3). Regardless of the surfactant employed, samples with a higher concentration of surfactant exhibited higher fluorescence emission intensity. This effect can be attributed to a higher curcumin concentration within the hydrophobic interior of the surfactant micelles, as well as less light scattering by any precipitated curcumin.

The majority of the curcumin-surfactant solutions were stable, i.e., the curcumin concentration remained constant throughout storage. The only exception was the curcumin-T80 solution below the CMC, which showed an appreciable decrease in absorbance and fluorescence intensity over time. In this system, extensive precipitation of curcumin was observed, which precluded measurement of the particle size at day 30 because a representative sample could not be obtained, and the particle size of the crystals was above the measurement range of the dynamic light scattering instrument. Overall, all the solutions produced with S465 had a smaller mean particle diameter than those produced with T80 (Table 1). There were no significant differences in the mean particle diameter among samples prepared with the same surfactant. Also, the storage of the stock solution for 30 days at 4 and 20 °C did not affect the mean particle diameter of the solutions (Table 1 and Figure S4). All samples produced with S465 or T80 had a relatively low charge, as indicated by their low ζ-potential, which can be attributed to the fact that these are nonionic surfactants. Moreover, there was no significant change in the ζ-potential values between days 0 and 30 storage for the majority of the samples (Table S1).

### 3.3. Influence of Surfactant Level on Microbial Photoinactivation 

**Figure 5:**
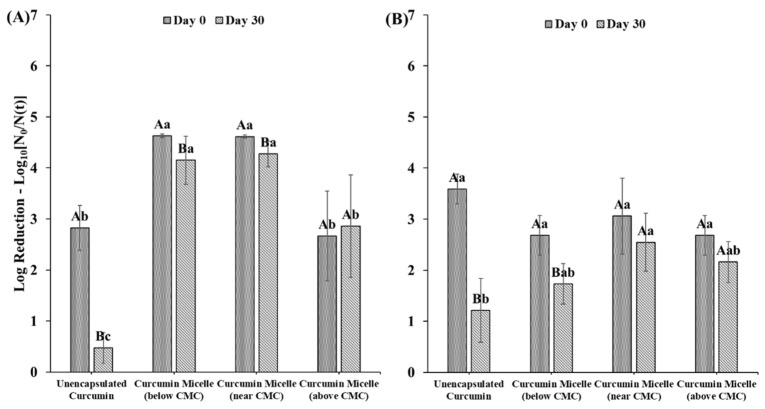
*E. coli* O157: H7 log reduction after photosensitization treatments with recently prepared (day 0) and stored (day 30) curcumin in solution or encapsulated in (**A**) S465 and (**B**) T80. Capital letters indicate differences between measurements at day 0 and 30. Lower-case letters indicate differences between treatments measured on the same day. Samples with the same letters are not significantly different (*p* > 0.05).

As mentioned earlier, curcumin-surfactant solutions with surfactant levels below, near, and above the CMCs were prepared; all contained 1 µM curcumin (5 mM sodium citrate buffer, pH 3.5). These samples were inoculated with the corresponding microorganisms and then irradiated for 5 min as described in Section 2.7. The efficacy of curcumin formulations that were freshly prepared or stored for 30 days was compared. The photoinactivation of the unencapsulated curcumin sample decreased significantly after storage. Conversely, photoinactivation by the curcumin-surfactant solution remained constant or only slightly decreased (Figure 5) after 30 days of storage. When S465 was present at concentrations below or near its CMC, photoinactivation was enhanced, regardless of the age of the stock solution. However, when S465 was present at concentrations above the CMC, the overall photoinactivation was lower than when using other curcumin-S465 solutions and similar to unencapsulated curcumin. This effect can be attributed to the fact that there were more surfactant micelles present that could solubilize the curcumin. As a result, a lower fraction of the curcumin partitioned into the cell walls of the bacteria, which reduced its ability to promote photoinactivation. This hypothesis is supported by the results shown in Figure 4, which indicated a higher fluorescence intensity for these systems, and is consistent with more of the curcumin being trapped in a highly restrictive environment, such as the hydrophobic core of the surfactant micelles [35,36]. When T80 was present below, near, and above the CMC, at day 0, the antimicrobial activity was similar to that of unencapsulated curcumin in terms of log reductions. But on day 30, due to the protective effect provided by the T80, the inactivation achieved was higher than the one observed using unencapsulated curcumin stored for 30 days. 

**Figure 6:**
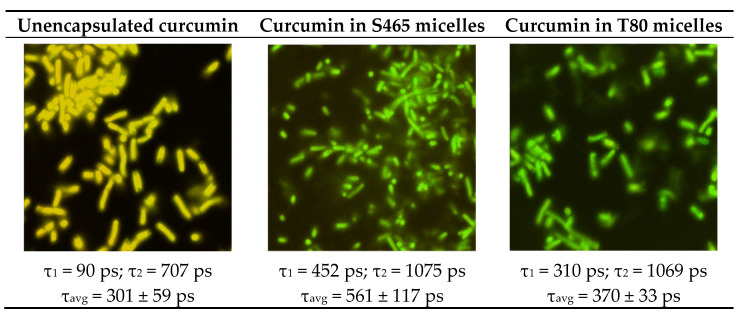
FLIM micrographs of *E. coli* O157: H7 treated with unencapsulated curcumin (**left**), in S465 (**middle**) and T80 micelles (**right**). The correspondent short and long components of the exponential fit of the lifetime are listed below each image along the average lifetime.

The analysis of the fluorescence lifetime micrographs provides some insights into the differential performance of the two surfactants (Figure 6). The micrographs for *E. coli* and unencapsulated curcumin exhibited shorter average fluorescence lifetimes than those containing curcumin and surfactant. A higher average lifetime suggests a more effective adsorption of the photosensitizer onto the microbial cell wall and in situ generation of ROS. The values of the average lifetimes are thereby consistent with the extent of inactivation obtained for each system.

### 3.4. Combined Effects on Microbial Photoinactivation

**Figure 7:**
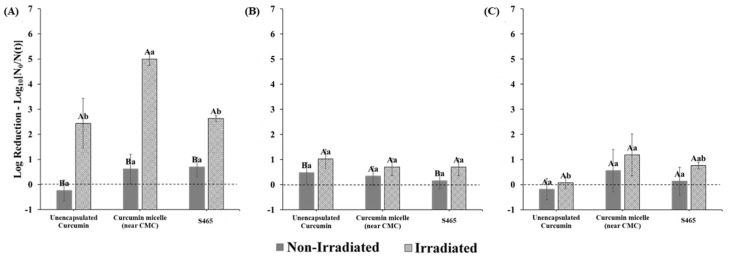
Reductions in *E. coli* O157: H7 counts after photosensitization with curcumin in solution and S465 micelles at (**A**) pH 3.5, (**B**) pH 5, and (**C**) pH 7.4. Capital letters indicate differences between irradiated and non-irradiated samples. Lower-case letters indicate differences within treatments (irradiated vs. non-irradiated). Means with same letters are not significantly different (*p* > 0.05).

**Figure 8:**
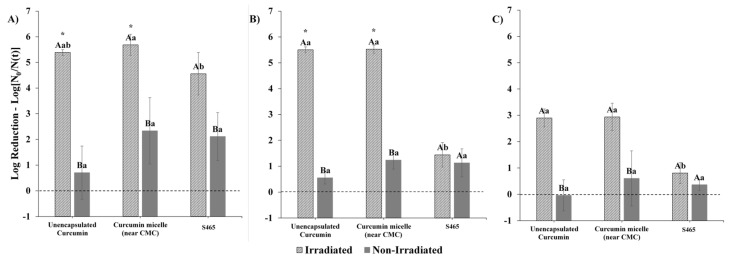
Reductions in *L. innocua* counts after photosensitization with curcumin in solution and S465 micelles at (**A**) pH 3.5, (**B**) pH 5, and (**C**) pH 7.4. “*” indicates that the MPN was below the limit of detection, and the log reduction was greater than 5. Capital letters indicate differences between irradiated and non-irradiated samples. Lower-case letters indicate differences within treatments (irradiated vs. non-irradiated). Means with same letters are not significantly different (*p* > 0.05).

In this series of experiments, we examined the contribution of each of the components in the curcumin-surfactant micellar solutions of the photoinactivation of *E. coli* O157:H7 and *L. innocua*. In this case, the surfactant concentrations used were close to the CMC values. Photoinactivation with curcumin-loaded micelles produced from S465 was highly affected by the pH of the system. The dependency of curcumin photosensitization activity on pH, albeit not within delivery systems, was also reported by de Oliveira et al. [21]. Similar to their findings, our study showed that at lower pH values (i.e., 3.5), there was a distinct enhancement in antimicrobial activity when both curcumin and S465 micelles were present (Figure 7). Interestingly, S465 micelles alone could reduce the number of viable *E. coli* O157:H7 cells to levels similar to those obtained with unencapsulated curcumin after irradiation. This observation also supports the reduction in the performance of curcumin-S465 systems when the surfactant concentration greatly exceeds the CMC. As mentioned earlier, under these conditions, the photoactivated antimicrobial activity of the curcumin is reduced because it is mainly trapped inside the hydrophobic interiors of the micelles, rather than in the microbial cells (Figure 5). Conversely, when the surfactant concentration is near (or below) the CMC, the antimicrobial activity may be enhanced because there are fewer surfactant micelles available to compete with the bacterial cells. It is not expected that the S465 itself contributes greatly to the antimicrobial activity through photosensitization because its absorbance and structural properties are not capable of producing high levels of ROS. However, it has been reported that surfactants can exhibit antimicrobial properties due to their ability to increase the permeability of cellular membranes [37,38]. In fact, a previous study indicated that micelles produced with Surfynol 485W, which has a similar structure to S465 (30 vs. 10 moles of ethylene oxide, respectively) and HLB values (18 vs. 13) could weakly inhibit the growth of *E. coli* O157:H7 and *Listeria monocytogenes* at low pH [39]. Gram-positive bacterium *L. innocua* was more sensitive than Gram-negative bacterium *E. coli* O157:H7 to photoinactivation by both the irradiated unencapsulated curcumin and the curcumin-loaded S465 micelles at all pH values (Figure 7 and Figure 8). A synergistic antimicrobial activity was not observed at pH 3.5 and pH 5 for *L. innocua* due to the limit of detection of the method used (Figure 8).

**Figure 9:**
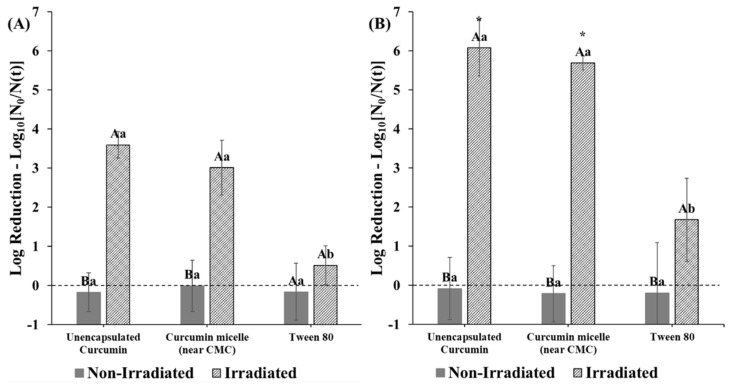
Reductions in (**A**) *E. coli* O157: H7 and (**B**) *L. innocua* counts photosensitized with curcumin in solution and T80 micelles at pH 3.5. “*” indicates that the log reduction was below the limit of detection of the method (MPN). Capital letters indicate differences between irradiated and non-irradiated samples. Lower-case letters indicate differences within treatments (irradiated vs. non-irradiated). Means with same letters are not significantly different.

When the photoinactivation of curcumin-loaded micelles produced from T80 was tested at pH 3.5, no synergistic antimicrobial activity between the curcumin and surfactant was observed. Unlike S465, using a delivery system produced by T80 did not further decrease *E. coli* O157:H7 counts present at pH 3.5 after irradiation (Figure 9). This may be due to the difference in the amount of surfactant required to produce the micelles and the molecular structure of the surfactants. Indeed, a previous study reported that the size of the headgroup affects the partitioning of nonionic surfactants into phospholipid liposomes, thereby causing differences in the rate of leakage of the contents inside them [38]. Similar to the effects obtained with S465 micelles, *L. innocua* was more susceptible to photoinactivation with curcumin-loaded T80 micelles than *E. coli* O157:H7 (Figure 9). 

*L. innocua* were more sensitive than *E. coli* O157:H7 to both types of curcumin-loaded micelles due to differences in the structure of their cellular membranes. Previous studies reported that Gram-positive bacteria are more vulnerable than Gram-negative to curcumin after irradiation [40,41]. Additionally, the use of delivery systems such as curcumin-loaded nanoparticles also contributed to an increased susceptibility in Gram-positive bacteria once irradiated with blue light [42]. Gram-positive bacteria have a peptidoglycan layer outside the cytoplasmic membrane, consisting of peptidoglycan, at least 40% by weight, and anionic polymers covalently linked to peptidoglycans [43]. However, this layer does not act as a permeability barrier as it is highly porous to small molecules [44]. Gram-negative bacteria have a more complex membrane structure. The outermost layer of Gram-negative bacteria is a membrane consisting of phospholipid bilayers embedded with lipopolysaccharide (LPS), outer membrane protein, and lipoprotein, which prevent substances such as antibiotics from entering the cytoplasm [44]. Therefore, the outer membrane of Gram-negative bacteria gives them some protection from photosensitization by inhibiting the diffusion of photosensitizers into the cells.

In summary, micellar delivery systems enhanced the photoactivated antimicrobial efficacy of curcumin. Micelles produced using S465 or T80 increased the stability and dispersibility and, consequently, the antimicrobial efficacy of curcumin in the aqueous phase over 30 days of storage. Micelles produced using S465, once diluted, had the best antimicrobial efficacy when the surfactant concentration was near or below the CMC because there was less partitioning of the curcumin between the hydrophobic domains in the micelles and the microbial cells. Also, there was a synergistic effect when both curcumin and S465 were present, which may have been due to the combined effect of curcumin and S465 on the cellular membrane of the bacteria.

Interestingly, the curcumin did not precipitate in the surfactant solutions even under conditions where the surfactant concentration was below the CMC. This effect may have been due to the ability of the surfactants to inhibit the nucleation and crystal growth of the curcumin. Presumably, the non-polar tails on the surfactant molecules could bind to the non-polar surfaces of the curcumin molecules, thereby preventing them from coming into close contact with each other.

## 4. Conclusions

The formation, stability, and photoinactivation efficacy of curcumin-surfactant solutions depend on the pH of the system, surfactant type, and the level of surfactant used. The addition of surfactants at any level to the curcumin solution enhanced its dispersibility, stability, and efficacy as a photosensitizer, thereby enhancing its antimicrobial activity. Surfactant addition also led to stable, concentrated curcumin solutions that could be diluted for use as surface sanitizers. All curcumin-surfactant solutions prepared from the two nonionic surfactants used (S465 or T80) were stable, and the encapsulated curcumin exhibited good photoinactivation after storage. However, only curcumin micelles with S465 concentrations below or near the CMC showed a synergistic antimicrobial activity between the curcumin and surfactant once irradiated, with this effect being most predominant at pH 3.5. This effect was mainly attributed to the stresses imposed on the cellular membranes by both of these compounds, which allowed increased influx of weak acids and curcumin into the microbial cells. Gram-positive bacteria, with less complex cellular membranes, were more susceptible than Gram-negative bacteria when curcumin-loaded micelles were used against them. Our data indicate that the use of a micelle-based delivery system facilitates adsorption and the generation of reactive oxygen species in the immediate environment of the microbial cell. Nevertheless, further studies are required to evaluate the underlying mechanisms of the synergistic microbial photoinactivation by photosensitizers and surfactants.

## Figures and Tables

**Table 1:** Mean diameters and polydispersity indexes (PDI) of stock curcumin/surfactant solutions during storage.

Curcumin/Surfactant Solution			Mean Particle Diameter Z-AverZage (nm) **				Polydispersity Index (PDI) **			
Curcumin Conc. (µM)	Surfactant		4 °C		20 °C		4 °C		20 °C	
	Type	Level	Day 0	Day 30	Day 0	Day 30	Day 0	Day 30	Day 0	Day 30
20	None	-	-	-	-	-	-	-	-	-
	S465	Below CMC	6.8 ± 2.1 ^Aa^	7.6 ± 2.2 ^Aa^	6.8 ± 2.1 ^Aa^	7.1 ± 1.8 ^Aa^	0.11 ± 0.06 ^Aa^	0.09 ± 0.08 ^Aa^	0.11 ± 0.06 ^Aa^	0.16 ± 0.06 ^Aa^
		Near CMC	5.8 ± 0.7 ^Aa^	5.3 ± 0.2 ^Aa^	5.8 ± 0.7 ^Aa^	7.1 ± 1.8 ^Aa^	0.11 ± 0.07 ^Aa^	0.06 ± 0.05 ^Aa^	0.11 ± 0.07 ^Aa^	0.08 ± 0.02 ^Aa^
		Above CMC	6.6 ± 1.8 ^Aa^	6.2 ± 0.7 ^Aa^	6.6 ± 1.8 ^Aa^	8.3 ± 2.3 ^Aa^	0.13 ± 0.03 ^Aa^	0.18 ± 0.03 ^Aa^	0.13 ± 0.03 ^Aa^	0.14 ± 0.01 ^Aa^
	T80	Below CMC *	-	-	-	-	-	-	-	-
		Near CMC	15 ± 1.0 ^Ab^	16 ± 5.3 ^Ab^	15 ± 1.0 ^Ab^	17 ± 2.8 ^Ab^	0.21 ± 0.09 ^Aa^	0.26 ± 0.08 ^Aa^	0.21 ± 0.09 ^Aa^	0.24 ± 0.07 ^Ab^
		Above CMC	15 ± 4.0 ^Ab^	19 ± 6.8 ^Ab^	15 ± 4.0 ^Ab^	17 ± 2.8 ^Ab^	0.29 ± 0.16 ^Aa^	0.33 ± 0.28 ^Aa^	0.29 ± 0.16 ^Aa^	0.23 ± 0.02 ^Ac^

* Samples too polydisperse to be measured with the Zetasizer NS. ** Capital letters were used to show differences between measurements at day 0 and 30; same letters indicate data was not significantly different (*p* > 0.05). Means with the same lower-case letters in the same column were not significantly different (*p* > 0.05).

## Data Availability

Data is contained within the article or Supplementary Material.

## Supplementary Materials

The following are available online at https://www.mdpi.com/article/10.3390/foods10081777/s1, Figure S1: Loading capacity of (A) Surfynol 465 and (B) T80 solution with curcumin dissolved in ethanol, Figure S2: Absorbance of curcumin in solution and encapsulated in (A) Surfynol 465 and (B) Tween 80 (T80) at different concentrations during storage at 4 °C for 30 days measured at λ = 425 nm, Figure S3: Normalized fluorescence intensity of curcumin in solution and encapsulated in (A) Surfynol 465 and (B) T80 at different concentrations during storage at 4 °C for 30 days, Figure S4: Average diameter of stock curcumin micelles produced with (A) Surfynol 465 and (B) T80 over 30-day storage at (1) 20 °C and (2) 4 °C, Table S1: Zeta-potential of stock micelle solution containing different types and amounts of surfactants.

## References

[1] Bulchholz A.L., Davidson G.R., Marks B.P., Todd E.C., Ryser E.T. (2012). Transfer of *Escherichia coli* O157:H7 from equipment surfaces to fresh-cut leafy greens during processing in a model pilot-plant production line with sanitizer-free water. J. Food Prot..

[2] McGlynn W. (2016). Guidelines for the Use of Chlorine Bleach as a Sanitizer in Food Processing Operations. Oklahoma Cooperative Extension Service. Division of Agricultural Sciences and Natural Resources. https://extension.okstate.edu/fact-sheets/guidelines-for-the-use-of-chlorine-bleach-as-a-sanitizer-in-food-processing-operations.html.

[3] Chen X., Hung Y.-C. (2017). Effects of organic load, sanitizer pH and initial chlorine concentration of chlorine-based sanitizers on chlorine demand of fresh produce wash waters. Food Control.

[4] Teng Z., Van Haute S., Zhou B., Hapeman C.J., Millner P.D., Wang Q., Luo Y. (2018). Impacts and interactions of organic compounds with chlorine sanitizer in recirculated and reused produce processing water. PLoS ONE.

[5] Thomas J.L., Palumbo M.S., Farrar J.A., Farver T.B., Cliver D.O. (2003). Industry practices and compliance with U.S. Food and Drug Administration Guidelines among California sprout firms. J. Food Prot..

[6] Hua Z., Korany A.M., El-Shinawy S.H., Zhu M.-J. (2019). Comparative evaluation of different sanitizers against *Listeria monocytogenes* biofilms on major food-contact surfaces. Front. Microbiol..

[7] Castano A.P., Demidova T.N., Hamblin M.R. (2004). Mechanisms in photodynamic therapy: Part one-Photosensitizers, photochemistry and cellular localization. Photodiagn. Photodyn. Ther..

[8] Min D.B., Boff J.M. (2002). Chemistry and reaction of singlet oxygen in foods. Compr. Rev. Food Sci. Food Saf..

[9] Abrahamse H., Hamblin M.R. (2016). New photosensitizers for photodynamic therapy. Biochem. J..

[10] Pouyet B., Chapelon R. (1987). Photochemical mechanisms in photosensitization. J. Phys. Colloq..

[11] Cossu M., Ledda L., Cossu A. (2021). Emerging trends in the photodynamic inactivation (P.D.I.) applied to the food decontamination. Food Res. Int..

[12] Hardwick J.P. (2015). Cytochrome P450 Function and Pharmacological Roles in Inflammation and Cancer.

[13] Macdonald I.J., Dougherty T.J. (2001). Basic principles of photodynamic therapy. J. Porphyr. Phthalocyanines.

[14] Schweitzer C., Schmidt R. (2003). Physical mechanisms of generation and deactivation of singlet oxygen. Chem. Rev..

[15] Dysart J.S., Patterson M.S. (2005). Characterization of photofrin photobleaching for singlet oxygen dose estimation during photodynamic therapy of MLL cells in vitro. Phys. Med. Biol..

[16] Luksiene Z., Peciulyte D., Jurkoniene S., Puras R. (2005). Inactivation of possible fungal food contaminants by photosensitization. Food Technol. Biotechnol..

[17] Buchovec I., Vaitonis Z., Luksiene Z. (2009). Novel approach to control *Salmonella enterica* by modern biophotonic technology: Photosensitization. J. Appl. Microbiol..

[18] Tønnesen H.H., Karlsen J. (1985). Studies on curcumin and curcuminoids. Z. Lebensm. Unters. Forsch..

[19] Karaffa L.S. (2013). The Merck Index: An Encyclopedia of Chemicals, Drugs, and Biologicals.

[20] Kharat M., Du Z., Zhang G., McClements D.J. (2017). Physical and chemical stability of curcumin in aqueous solutions and emulsions: Impact of pH, temperature, and molecular environment. J. Agric. Food Chem..

[21] De Oliveira E.F., Tikekar R., Nitin N. (2018). Combination of aerosolized curcumin and UV-A light for the inactivation of bacteria on fresh produce surfaces. Food Res. Int..

[22] Hiemenz P.C., Rajagopalan R. (2016). Principles of Colloid and Surface Chemistry, Revised and Expanded.

[23] Duan Y., Wang J., Yang X., Du H., Xi Y., Zhai G. (2015). Curcumin-loaded mixed micelles: Preparation, optimization, physicochemical properties and cytotoxicity in vitro. Drug Deliv..

[24] Leung M.H., Colangelo H., Kee T.W. (2008). Encapsulation of curcumin in cationic micelles suppresses alkaline hydrolysis. Langmuir.

[25] Wang X., Gao Y. (2018). Effects of length and unsaturation of the alkyl chain on the hydrophobic binding of curcumin with Tween micelles. Food Chem..

[26] Uluata S., McClements D.J., Decker E.A. (2016). Riboflavin-induced oxidation in fish oil-in-water emulsions: Impact of particle size and optical transparency. Food Chem..

[27] Food and Drug Administration (FDA) (2017). Bacteriological Analytical Manual (BAM).

[28] Colaruotolo L.A., Peters E., Corradini M.G. (2021). Novel luminescent techniques in aid of food quality, product development, and food processing. Curr. Opin. Food Sci..

[29] Miraglia D.B., Rodríguez J.L., Minardi R.M., Schulz P.C. (2011). Critical micelle concentration and HLB of the sodium oleate–hexadecyltrimethylammonium bromide mixed system. J. Surfactants Deterg..

[30] Komaiko J.S., McClements D.J. (2016). Formation of food-grade nanoemulsions using low-energy preparation methods: A review of available methods. Comp. Rev. Food Sci. Food Saf..

[31] Gaysinsky S. (2004). Physicochemical and Antimicrobial Properties of Antimicrobials Encapsulated in Surfactant-Based Nanoparticles. Master’s Thesis.

[32] Ma P., Zeng Q., Tai K., He X., Yao Y., Hong X., Yuan F. (2018). Development of stable curcumin nanoemulsions: Effects of emulsifier type and surfactant-to-oil ratios. J. Food Sci. Technol..

[33] Páhi A.B., Király Z., Puskás S. (2009). Mass spectrometric characterization of the nonionic Gemini surfactant Surfynol 465 and a microcalorimetric study of its micelle formation in water. Colloids Surf. A.

[34] Sato S., Kishimoto H., Mittal K.L. (1989). Behavior of nonionic surfactant, Surfynol 465, in aqueous media. Surfactants in Solution.

[35] Hammer K.A., Carson C.F., Riley T.V. (1999). Influence of organic matter, cations and surfactants on the antimicrobial activity of *Melaleuca alternifolia* (tea tree) oil in vitro. J. Appl. Microbiol..

[36] Uzhinov B.M., Ivanov V.L., Melnikov M.Y. (2011). Molecular rotors as luminescent sensors of local viscosity and viscous flow in solution and organized systems. Russ. Chem. Rev..

[37] Le Maire M., Moeller J.V., Champeil P. (1987). Binding of a nonionic detergent to membranes: Flip-flop rate and location on the bilayer. Biochemistry.

[38] De La Maza A., Parra J., Garcia M., Ribosa I., Leal J.S. (1992). Permeability changes in the phospholipid bilayer caused by nonionic surfactants. J. Colloid Interface Sci..

[39] Gaysinsky S., Davidson P.M., Bruce B.D., Weiss J. (2005). Stability and antimicrobial efficiency of eugenol encapsulated in surfactant micelles as affected by temperature and pH. J. Food Prot..

[40] Dahl T.A., Mcgowan W.M., Shand M.A., Srinivasan V.S. (1989). Photokilling of bacteria by the natural dye curcumin. Arch. Microbiol..

[41] De Oliveira E.F., Tosati J.V., Tikekar R.V., Monteiro A.R., Nitin N. (2018). Antimicrobial activity of curcumin in combination with light against *Escherichia coli* O157: H7 and *Listeria innocua*: Applications for fresh produce sanitation. Postharvest Biol. Technol..

[42] Agel M.R., Baghdan E., Pinnapireddy S.R., Lehmann J., Schäfer J., Bakowsky U. (2019). Curcumin loaded nanoparticles as efficient photoactive formulations against Gram-positive and Gram-negative bacteria. Colloids Surf. B.

[43] Mozes N. (1991). Microbial Cell Surface Analysis.

[44] Maisch T., Szeimies R.-M., Jori G., Abels C. (2004). Antibacterial photodynamic therapy in dermatology. Photochem. Photobiol. Sci..

