# Supplementary material for: Use of Micellar Delivery Systems to Enhance Curcumin’s Stability and Microbial Photoinactivation Capacity
Source: Foods. 2021 Jul 31;10(8):1777. doi: 10.3390/foods10081777 (PMC8394612; doi:10.3390/foods10081777)
Supplement: Supplementary file 1 [file foods-10-01777-s001.zip › foods-1289885-supplementary.pdf]

Supplementary Material

Use of micellar delivery to enhance curcumin's stability and microbial photoinactivation

Figure S1

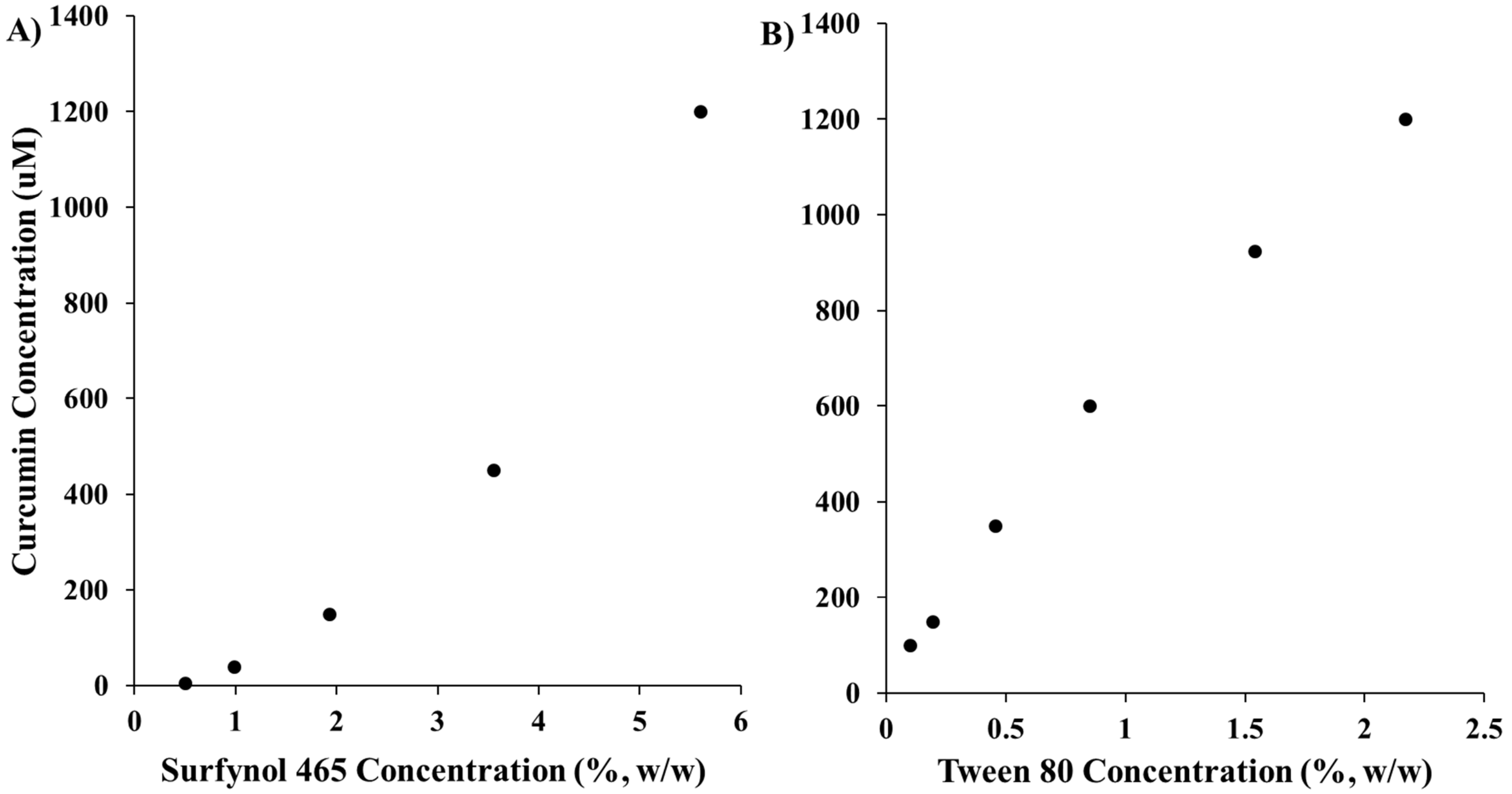

Figure S1. Loading capacity of A) Surfynol 465 and B) T80 solution with curcumin dissolved in ethanol.

Figure S2

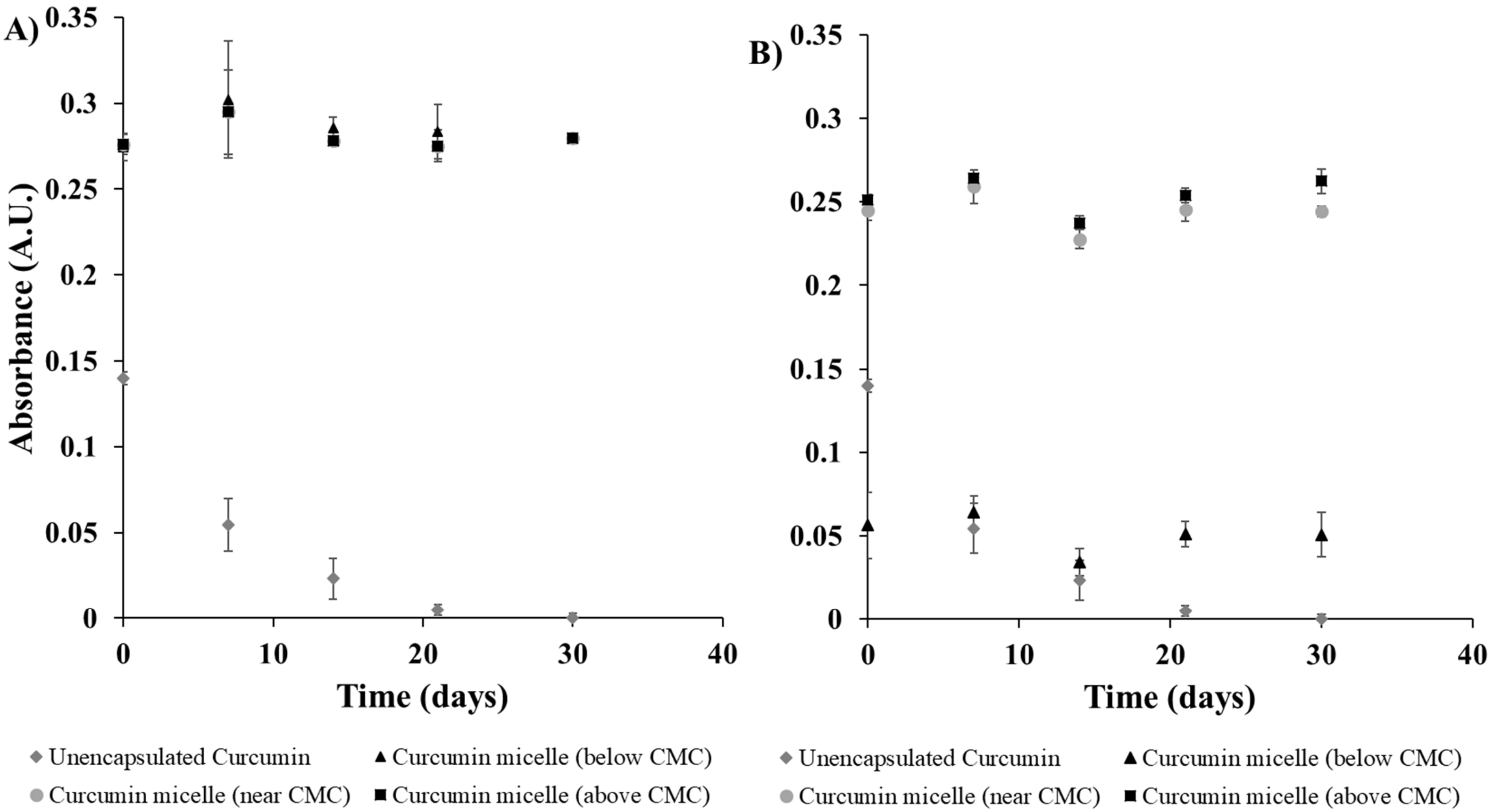

Figure S2. Absorbance of curcumin in solution and encapsulated in A) Surfynol 465 and B) Tween 80 (T80) at different concentrations during storage at 4 °C for 30 days measured at λ = 425 nm.

Figure S3

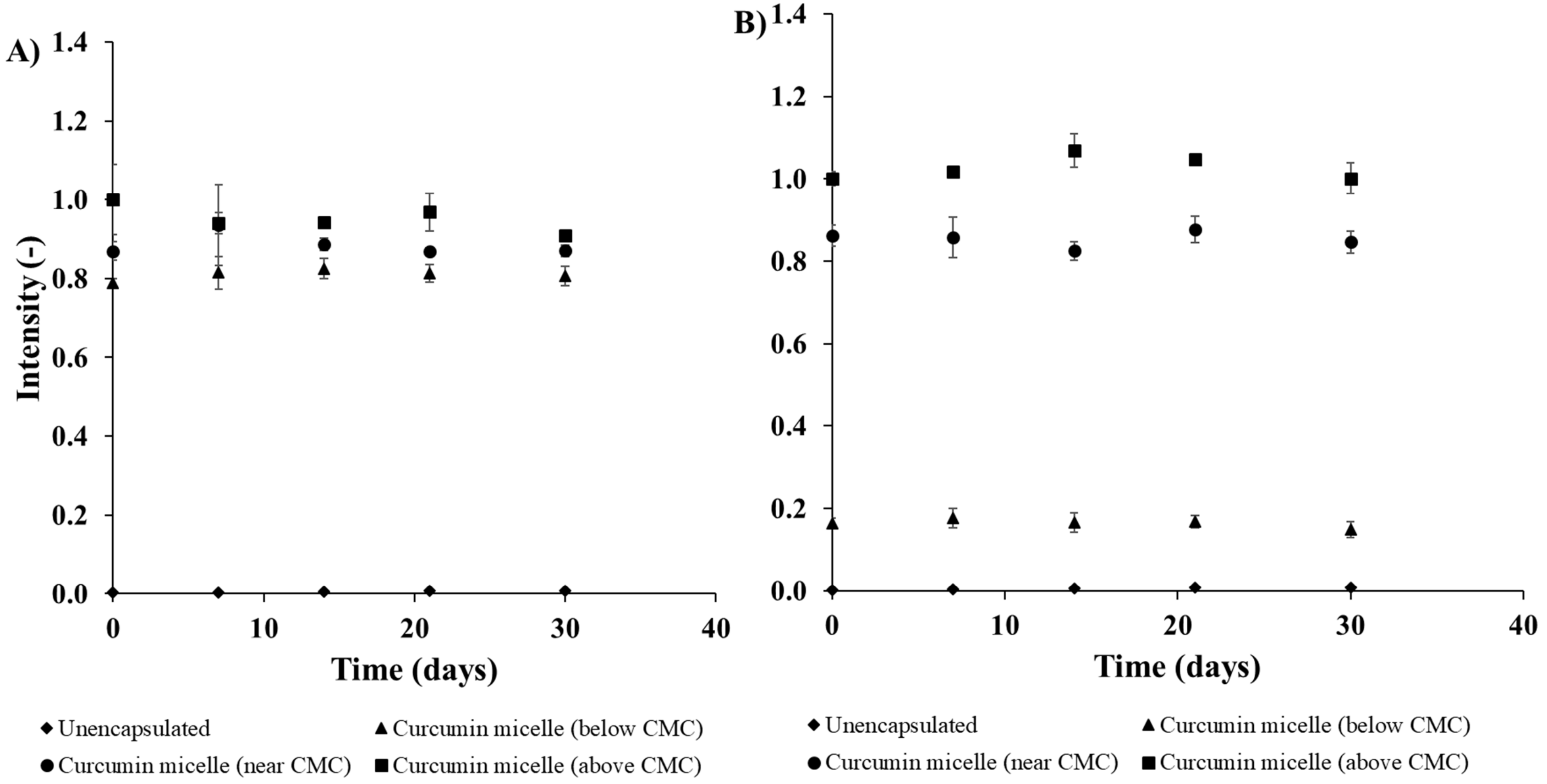

Figure S3. Normalized fluorescence intensity of curcumin in solution and encapsulated in A) Surfynol 465 and B) T80 at different concentrations during storage at 4 °C for 30 days.

Figure S4

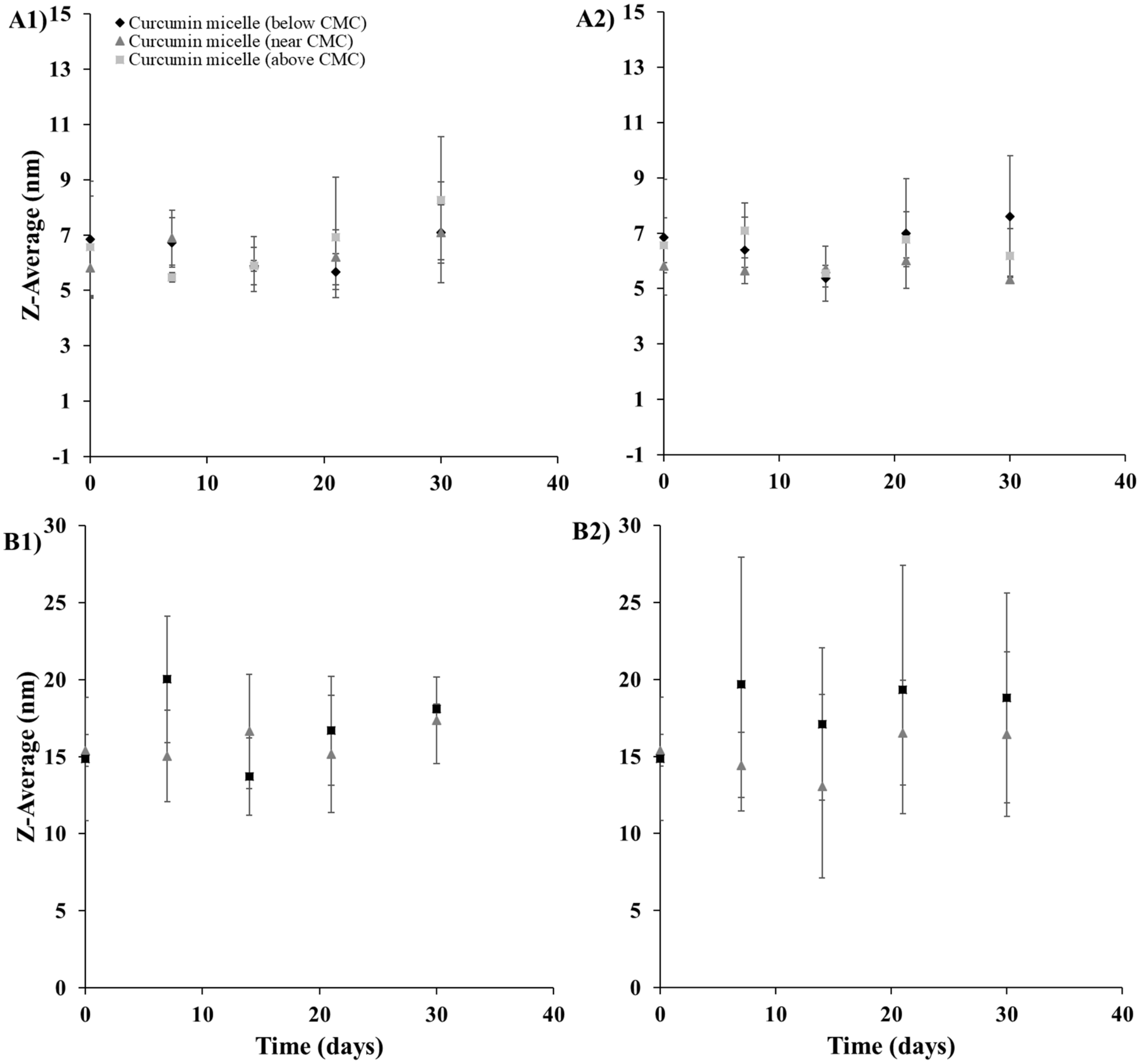

Figure S4. Average diameter of stock curcumin micelles produced with A) Surfynol 465 and B) T80 over 30-day storage at 1) 20°C and 2) 4°C

Table S1. Zeta-potential of stock micelle solution containing different types and amounts of surfactants

Curcumin Micelle			Mean Particle Diameter Z-average (nm)			
			4°C		20°C	
**Curcumin Conc. (µM)**	Surfactant Type	Surfactant Level	Day 0	Day 30	Day 0	Day 30
**20**	Surfynol 465	Below CMC	-0.3±0.3^{A}[2]	-0.4±0.5^{A}	-0.3±0.3^{A}[2]	-0.3±0.2^{A}
		Near CMC	-0.02±0.9^{A}	-0.2±0.1^{A}	-0.4±0.7^{A}	-0.3±0.8^{A}
		Above CMC	-0.4±0.2^{A}	0.003±0.4^{A}	-0.4±0.2^{A}	-0.3±0.3^{A}
	Tween 80	Below CMC	-[1]	-	-	-
		Near CMC	-1.3±1.6^{A}	-0.2±0.4^{B}	-1.3±1.6^{A}	-0.5±1.8^{A}
		Above CMC	-0.2±0.5^{A}	-0.1±0.05^{A}	-0.2±0.5^{A}	-0.1±0.4^{A}

[1] Samples too polydisperse to be measured with the Zetasizer NS due to the formation of curcumin crystals.

[2] Capital letters were used to show differences between measurements at day 0 and 30; same letters indicate data was not significantly different (p>.05)
